# Full-Length Genome of an *Ogataea polymorpha* Strain CBS4732 *ura3*Δ Reveals Large Duplicated Segments in Subtelomeric Regions

**DOI:** 10.3389/fmicb.2022.855666

**Published:** 2022-04-04

**Authors:** Jia Chang, Jinlong Bei, Qi Shao, Hemu Wang, Huan Fan, Tung On Yau, Wenjun Bu, Jishou Ruan, Dongsheng Wei, Shan Gao

**Affiliations:** ^1^Key Laboratory of Molecular Microbiology and Technology, Ministry of Education, College of Life Science, Nankai University, Tianjin, China; ^2^Agro-Biological Gene Research Center, Guangdong Academy of Agricultural Sciences, Guangdong Provincial Key Laboratory for Crop Germplasm Resources Preservation and Utilization, Guangzhou, China; ^3^Guangdong Laboratory for Lingnan Modern Agriculture, Guangzhou, China; ^4^Tianjin Hemu Health Biotechnological Co., Ltd., Tianjin, China; ^5^Tianjin Institute of Animal Husbandry and Veterinary Research, Tianjin, China; ^6^John Van Geest Cancer Research Centre, School of Science and Technology, Nottingham Trent University, Nottingham, United Kingdom; ^7^Department of Rural Land Use, Scotland’s Rural College, Aberdeen, United Kingdom; ^8^School of Mathematical Sciences, Nankai University, Tianjin, China

**Keywords:** methylotrophic yeast, *Hansenula polymorpha*, rDNA quadruple, genome expansion, long terminal repeat

## Abstract

**Background:**

Currently, methylotrophic yeasts (e.g., *Pichia pastoris*, *Ogataea polymorpha*, and *Candida boindii*) are subjects of intense genomics studies in basic research and industrial applications. In the genus *Ogataea*, most research is focused on three basic *O. polymorpha* strains-CBS4732, NCYC495, and DL-1. However, the relationship between CBS4732, NCYC495, and DL-1 remains unclear, as the genomic differences between them have not be exactly determined without their high-quality complete genomes. As a nutritionally deficient mutant derived from CBS4732, the *O. polymorpha* strain CBS4732 *ura3*Δ (named HU-11) is being used for high-yield production of several important proteins or peptides. HU-11 has the same reference genome as CBS4732 (noted as HU-11/CBS4732), because the only genomic difference between them is a 5-bp insertion.

**Results:**

In the present study, we have assembled the full-length genome of *O. polymorpha* HU-11/CBS4732 using high-depth PacBio and Illumina data. Long terminal repeat retrotransposons (LTR-rts), rDNA, 5′ and 3′ telomeric, subtelomeric, low complexity and other repeat regions were exactly determined to improve the genome quality. In brief, the main findings include complete rDNAs, complete LTR-rts, three large duplicated segments in subtelomeric regions and three structural variations between the HU-11/CBS4732 and NCYC495 genomes. These findings are very important for the assembly of full-length genomes of yeast and the correction of assembly errors in the published genomes of *Ogataea* spp. HU-11/CBS4732 is so phylogenetically close to NCYC495 that the syntenic regions cover nearly 100% of their genomes. Moreover, HU-11/CBS4732 and NCYC495 share a nucleotide identity of 99.5% through their whole genomes. CBS4732 and NCYC495 can be regarded as the same strain in basic research and industrial applications.

**Conclusion:**

The present study preliminarily revealed the relationship between CBS4732, NCYC495, and DL-1. Our findings provide new opportunities for in-depth understanding of genome evolution in methylotrophic yeasts and lay the foundations for the industrial applications of *O. polymorpha* CBS4732, NCYC495, DL-1, and their derivative strains. The full-length genome of *O. polymorpha* HU-11/CBS4732 should be included into the NCBI RefSeq database for future studies of *Ogataea* spp.

## Introduction

Currently, methylotrophic yeasts (e.g., *Pichia pastoris*, *Hansenula polymorpha*, and *Candida boindii*) are subjects of intense genomics studies in basic research and industrial applications. However, genomic research on *Ogataea (Hansenula) polymorpha* trails behind that on *P. pastoris* (Ravin et al., 2013), although they both are widely used species of methylotrophic yeasts. In the genus *Ogataea*, most research is focused on three basic *O. polymorpha* strains—CBS4732 (synonymous to NRRL Y-5445 or ATCC34438), NCYC495 (synonymous to NRRL Y-1798, ATCC14754, or CBS1976), and DL-1 (synonymous to NRRL Y-7560 or ATCC26012). These three strains are of independent geographic and ecological origins: CBS4732 was originally isolated from soil irrigated with waste water from a distillery in Pernambuco, Brazil in 1959 (Morais and Maia, 1959); NCYC495 is identical to a strain first isolated from spoiled concentrated orange juice in Florida and initially designated as *Hansenula angusta* by Wickerham (1951); DL-1 was isolated from soil by Levine and Cooney (1973). CBS4732 and its derivatives—LR9 and RB11—have been developed as genetically engineered strains to produce many heterologous proteins, including enzymes (e.g., feed additive phytase), anticoagulants (e.g., hirudin and saratin), and an efficient vaccine against hepatitis B infection (Massoud et al., 2003). As a nutritionally deficient mutant derived from CBS4732, the *O. polymorpha* strain HU-11 (CBS4732 *ura3*Δ) (Wang et al., 2007) is being used for high-yield production of several important proteins or peptides, particularly including recombinant hepatitis B surface antigen (HBsAg) vaccine (Wang H. et al., 2016) and hirudin (Wang et al., 2011). HU-11 has the same reference genome as CBS4732 (noted as HU-11/CBS4732), as the only genomic difference between them is a 5-bp insertion caused by frame-shift mutation of its *URA3* gene, which encodes orotidine 5′-phosphate decarboxylase. Although CBS4732 and NCYC495 are classified as *O. polymorpha*, and DL-1 is reclassified as *O. parapolymorpha* (Hanson et al., 2017), the relationship between CBS4732, NCYC495, and DL-1 remains unclear, as the genomic differences between them have not been exactly determined due to lack of their high-quality complete genomes. Thus, knowledge obtained from any of the three strains can’t be used to investigate the other two strains.

To facilitate genomic research of yeasts, genome sequences have been increasingly submitted to the Genome-NCBI datasets. Among the genomes of 34 species in the *Ogataea* or *Candida* genus (Supplementary File 1), those of NCYC495 and DL-1 have been assembled at chromosome levels. However, the other genomes have been assembled at contig or scaffold levels. Furthermore, the genome sequence of CBS4732 was not available in the Genome-NCBI datasets until this manuscript was drafted. Among the genomes of 33 *Komagataella* (*Pichia*) spp., the genome of the *P. pastoris* strain GS115 is the only genome assembled at the chromosome level. The main problem of the *Ogataea*, *Candida*, and *Pichia* genome data is their incomplete sequences and poor annotations. For example, the rDNA sequence (GenBank: FN392325) of *P. pastoris* GS115 cannot be well aligned to its genome (GenBank assembly: GCA_001708105). Most genome sequences do not contain complete subtelomeric regions and, as a result, subtelomeres are often overlooked in comparative genomics (Brown, 2010). For example, the genome of DL-1 has been analyzed for better understanding the phylogenetics and molecular basis of *O. polymorpha* (Ravin et al., 2013); however, it does not contain complete subtelomeric regions due to assembly using short sequences. Another problem of the *Ogataea*, *Candida*, and *Pichia* genome data is that the mitochondrial (mt) genome sequence is not simultaneously released with the corresponding nuclear genome sequences. The only exception is the *O. polymorpha* DL-1 mt genome (RefSeq: NC_014805). Therefore, more complete genome sequences of *Ogataea* spp. are being accomplished to bridge the gap between basic research and industrial applications. For example, a new project has been conducted to provide a high-quality complete genome of DL-1 (GenBank: CP080316-22) based on Nanopore technology.

In the present study, we have assembled the full-length genome of *O. polymorpha* HU-11/CBS4732 using high-depth PacBio and Illumina data, and conducted the annotation and analysis to achieve the following research goals: (1) to provide a high-quality and well-curated reference genome for future studies of *Ogataea* spp.; (2) to determine the relationship between CBS4732, NCYC495, and DL-1; and (3) to discover important genomic features (e.g., high yield) of *Ogataea* spp. for basic research (e.g., synthetic biology) and industrial applications.

## Results and Discussion

### Genome Sequencing, Assembly and Annotation

**FIGURE 1:**
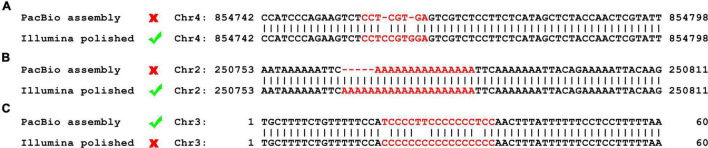
Errors in PacBio data and Illumina data. The errors in the low complexity and short tandem repeat (STR) regions can be corrected during the genome polishing using Illumina data, while the errors in the long (>10 G or C) poly(GC) regions need be curated using PacBio data after the genome polishing. **(A)** An example to show that the assembled genomes using high-depth PacBio data still contain errors in the low complexity regions. **(B)** An example to show that the assembled genomes using high-depth PacBio data still contain errors in the STR regions. **(C)** An example to show that the genome polishing using Illumina data causes errors in the long poly (GC) regions.

One 500 bp and one 10 Kbp DNA library were prepared using fresh cells of *O. polymorpha* HU-11 and sequenced on the Illumina HiSeq X Ten and PacBio Sequel platforms, respectively, for *de novo* assembly of a high-quality genome. Firstly, 18,319,084,791 bp cleaned PacBio DNA-seq data were used to assembled the complete genome, except the rDNA region, with an extremely high depth of ∼1800X. However, the draft genome using high-depth PacBio data still contained two types of errors in the low complexity (Figure 1A) and the short tandem repeat (STR) regions, respectively (Figure 1B). Then, 6,628,480,424 bp cleaned Illumina DNA-seq data were used to polish the complete genome of HU-11/CBS4732 to remove the two types of errors. However, Illumina DNA-seq data contained errors in the long (>10 G or C) poly(GC) regions. Following this, the poly(GC) regions, polished using Illumina DNA-seq data, were curated using PacBio subreads (Figure 1C). Finally, 5′ and 3′ telomeric, subtelomeric, rDNA, Long Terminal Repeat retrotransposons (LTR-rts), low complexity, and other repeat regions were exactly determined and confirmed by human curation (see section “Materials and Methods”). The complete *O. polymorpha* HU-11/CBS4732 genome is a full-length genome, which is defined to has sequences ending at the 5′ and 3′ telomeric sites without gaps and ambiguous nucleotides (Xu et al., 2020).

**FIGURE 2:**
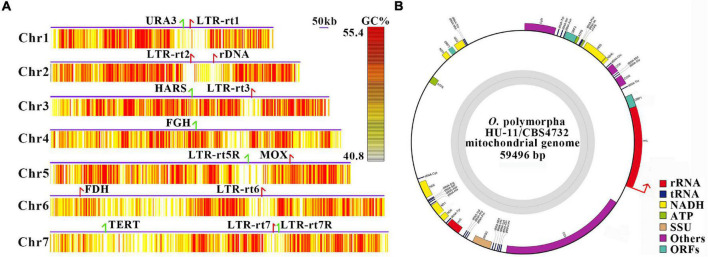
Full-length genome of *Ogataea polymorpha* HU-11/CBS4732. **(A)** The full-length *O. polymorpha* HU-11/CBS4732 genome includes the complete sequences of seven linear chromosomes, which were named as 1–7 from the smallest to the largest. The 5′ and 3′ telomeric regions were not included. The minimum, Q_90_, Q_75_, Q_50_, Q_25_, Q_10_, and maximum of GC contents (%) are 0.08, 0.408 0.436, 0.472, 0.514, 0.554, and 0.732. The GC contents (%) were calculated by 500-bp sliding windows and then trimmed between Q_10_ and Q_90_ for plotting the heatmaps. Long terminal repeat retrotransposons (LTR-rts) and markers genes are indicated by arrows (red and green colors represent sense and antisense strands) in the chromosomes. Markers genes of chromosome 1–7 include URA3 (encoding orotidine 5′-phosphate decarboxylase), rDNA, HARS (Hansenula autonomously replicating sequence), FGH (S-formylglutathione hydrolase), MOX (methanol oxidase), FDH (Formate dehydrogenase) and TERT (telomerase reverse transcriptase), respectively. **(B)** For the data submission to the GenBank database, the genome sequence of circular mitochondrion was anticlockwise linearized, starting at the first nt (indicated by a red arrow) of rrnL, which may include a part of the control region. SSU, small subunit; RPS3, ribosomal protein S3; rrnL, large subunit ribosomal RNA; rrnS, small subunit ribosomal RNA.

The full-length *O. polymorpha* HU-11/CBS4732 genome includes the complete sequences of all seven chromosomes (Figure 2A), which were named as 1 to 7 from the smallest to the largest, respectively (Table 1). As the 5′ and 3′ telomeric regions vary in lengths, they were not included into the seven linear chromosomes of HU-11/CBS4732. Analysis of long PacBio subreads revealed that the telomeric regions at 5′ and 3′ ends of each chromosome consist of tandem repeats (TRs) [ACCCCGCC]_*n*_ and [GGCGGGGT]_*n*_ (*n* is the copy number) with average lengths of 166 bp and 168 bp (∼20 copy numbers), respectively. Finally, we released the data of the nuclear genome (GenBank: CP073033-39) with a summed sequence length of 9.1 Mbp and the mt genome (GenBank: CP073040) with a sequence length of 59,496 bp (Table 1). For the submission to the GenBank database, the sequence of circular mt genome (Figure 2B) was anticlockwise linearized, starting at the first nt of large subunit ribosomal RNA (rrnL). The complete genome sequence of the *O. polymorpha* strain CBS4732 ura3Δ (named HU-11) is available at the NCBI GenBank database, which need be included into the NCBI RefSeq database to facilitate future studies on *O. polymorpha* CBS4732 and its derivatives- LR9, RB11, and HU-11.

**TABLE 1:** Genomes of three basic *Ogataea polymorpha* strains.

Chromosome	CBS4732/HU-11	NCYC495	DL-1	HU-11 Size (bp)	Marker
Chr1	CP073033	NW_017264703	NC_027865	1,000,895	URA3
Chr2	CP073034	NW_017264704	NC_027866	1,125,341	rDNA
Chr3	CP073035	NW_017264702	NC_027864	1,265,401	HARS
Chr4	CP073036	NW_017264701	NC_027863	1,315,956	FGH
Chr5	CP073037	NW_017264700	NC_027862	1,357,435	MOX
Chr6	CP073038	NW_017264698	NC_027860	1,513,391	FDH
Chr7	CP073039	NW_017264699	NC_027861	1,525,912	TERT
ChrM	CP073040	NA	NC_014805	59,496	COIII
Total (Mbp)	9.1	8.97	8.87		
GC%	47.76	47.86	47.83		
Gene^#^	5453*	5454*	5309*		
tRNA^#^	80	80	80		
rRNA^#^	4 × 20	4 × 6	4 × 25		

HU-11 has the same reference genome as CBS4732 (noted as HU-11/CBS4732), because the only genomic difference between them is a 5-bp insertion. ^#^The numbers of mitochondrial genes in the LTR-rts were not counted. *The genome sequences of NCYC495 and DL-1 with annotations were corrected, so the numbers of protein-coding genes (>150 bp) are different from their original records. The full-length O. polymorpha HU-11/CBS4732 genome includes the complete sequences of all seven chromosomes, which were named as 1–7 from the smallest to the largest, respectively. As the 5′ and 3′ telomeric regions vary in lengths, they were not included into the seven linear chromosomes of HU-11/CBS4732. The accession numbers of NCYC495 and DL-1 were mapped to the chromosome numbers of HU-11, according to the marker genes of seven chromosomes. They are: URA3 (encoding orotidine 5′-phosphate decarboxylase), rDNA, HARS(Hansenula autonomously replicating sequence), FGH (S-formylglutathione hydrolase), MOX(methanol oxidase), FDH (Formate dehydrogenase), TERT (telomerase reverse transcriptase) and COIII (cytochrome c oxidase subunit 3).

The HU-11/CBS4732 (nuclear) genome has a summed length of 9.1 Mbp that is close to the estimated length of the *O. polymorpha* DL-1 genome (Ravin et al., 2013), while the NCYC495 (RefSeq: NW_017264698-704) and DL-1 genomes (RefSeq: NC_027860-66) have shorter lengths of 8.97 and 8.87 Mbp, respectively (Table 1), as both of them are incomplete and have many errors at 5′ and 3′ ends of their chromosomes (Supplementary File 1). The GC contents of the HU-11, NCYC495, and DL-1 genomes are very close (∼48%). Syntenic comparison (see section “Materials and Methods”) revealed that *O. polymorpha* HU-11/CBS4732 is so phylogenetically close to NCYC495 that the syntenic regions cover nearly 100% of their genomes, however, HU-11/CBS4732 and NCYC495 are significantly distinct from DL-1. Then, we discovered large duplicated segments (LDSs) in the subtelomeric regions and exactly determined all the structural variations (SVs) between HU-11/CBS4732 and NCYC495 (Detailed later). We improved the annotations of NCYC495 protein-coding genes (>150 bp) using a high quality RNA-seq data of NCYC495 (NCBI SRA: SRP124832), and then HU-11/CBS4732 and DL-1 genes by gene ID mapping (Supplementary File 2). As a result, we updated the annotations (Table 1) of: (1) 5,453 protein-coding genes of HU-11/CBS4732, including 5,021 single-exon genes, and 432 multi-exon genes; (2) 5,454 protein-coding genes of NCYC495, including 5,022 single-exon genes, and 432 multi-exon genes; (3) 5,309 protein-coding genes of DL-1, including 4,843 single-exon genes, and 464 multi-exon genes; and (4) 80 identical tRNA genes of HU-11/CBS4732, NCYC495, and DL-1. For 432 multi-exon genes of HU-11/CBS4732, only the longest splicing isoforms of them were annotated. Then, 5,453 CDSs (Supplementary File 3) were identified from 5,453 genes of HU-11/CBS4732. Furthermore, only 13 single-exon genes of HU-11/CBS4732 or NCYC495 were not detected to be expressed using the RNA-seq data SRP124832, while 87 genes (data not shown) of DL-1 have been reported to be not expressed in the previous study (Ravin et al., 2013).

### Organization of rDNA Genes

**FIGURE 3:**
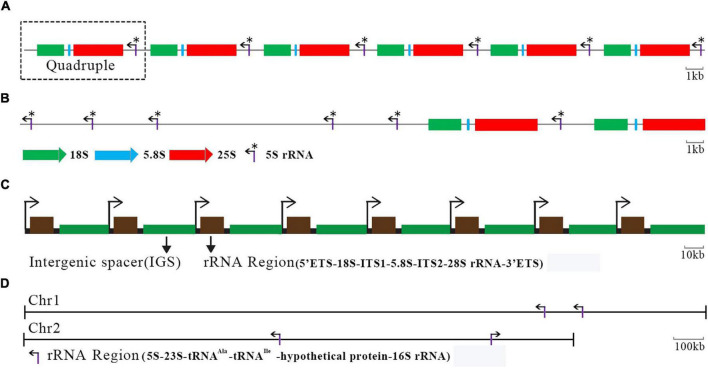
Organization of rDNA genes in yeasts, human and bacteria. **(A)** The only rDNA locus is in chromosome 2 (GenBank: CP073034) of the *Ogataea polymorpha* HU-11/CBS4732 genome, containing 20 copies of tandem repeats (TRs). Here only six copies of TRs are shown. **(B)** An rDNA TR of Saccharomyces cerevisiae also contains 5S, 18S, 5.8S, and 25S rDNAs as a quadruple, repeating 2 times in chromosome 7 of its genome. Four other 5S rDNAs are located separately away from the rDNA quadruples in *S. cerevisiae*. **(C)** Each human rDNA unit has an rRNA region and an intergenic spacer (IGS). Here only eight units are shown. ITS, internal transcribed spacer; ETS, external transcribed spacers. **(D)** There are four copies of rRNA regions at two rDNA loci in chromosome 1 (GenBank: CP022603) and 2 (GenBank: CP022604) of the *Ochrobactrum quorumnocens* genome. *Indicate the 5S rRNA.

An rDNA TR of HU-11/CBS4732, NCYC495, or DL-1 encodes 5S, 18S, 5.8S, and 25S rRNAs (named as quadruple in the present study), with a length of 8,145 bp (Supplementary File 1). As the largest TR region (∼162 Kbp) in the HU-11/CBS4732 genome, the only rDNA locus is in chromosome 2. The organization of rDNA TRs is conserved in the *Ogataea* genus. TRs of HU-11/CBS4732 and NCYC495 rDNAs share a very high nucleotide (nt) sequence identity of 99.5% (8,115/8,152), while TRs of HU-11/CBS4732 and DL-1 rDNAs share a comparatively low nt sequence identity of 97% (7,530/7,765). The major difference of rDNAs among *Ogataea* spp. lie in their copy numbers. The copy number of rDNA TRs was estimated as 20 in the HU-11/CBS4732 genome (Figure 3A), while that was estimated as 6 and 25 in NCYC495 and DL-1, respectively (Ravin et al., 2013). An rDNA TR of *Saccharomyces cerevisiae* also contains 5S, 18S, 5.8S, and 25S rDNAs as a quadruple, repeating two times in chromosome 7 of its genome (Figure 3B), however, four other 5S rDNAs are located separately away from the rDNA quadruples in *S. cerevisiae*. Different from *O. polymorpha* or *S. cerevisiae* with only one rDNA locus, *Pichia pastoris* GS115 carries several rDNA loci, which are interspersed in three of its four chromosomes. Since the genome of *P. pastoris* GS115 (GenBank assembly: GCA_001708105) is incomplete and poorly annotated, we estimated the copy number of its rDNAs as three or more. In animals, rDNAs encoding 18S, 5.8S, and 28S rRNAs are also organized in TRs and transcribed into a single RNA precursor by RNA polymerase I. As a typical example, human has approximately 200–600 rDNA copies (Figure 3C) distributed in short arms of the five acrocentric chromosomes (chromosomes 13, 14, 15, 21, and 22) (Agrawal and Ganley, 2018). In prokaryotic cells, 5S, 23S, and 16S rRNA genes are typically organized as a co-transcribed operon. There may be one or more copies of the operon dispersed in the genome and the copy numbers typically range from 1 to 15 in bacteria. As a typical example, *Ochrobactrum quorumnocens* has four copies of co-transcribed operons at two rDNA loci (Figure 3D) in chromosome 1 (GenBank: CP022603) and 2 (GenBank: CP022604). Compared to *S. cerevisiae*, human, and *O. quorumnocens* rDNAs (Figures 3B–D), the rDNAs of *O. polymorpha* are more closely organized. By the organization in TR, 20 copies of *O. polymorpha* rDNA quadruples are transcribed in a large (>162 Kbp) co-transcribed operon, suggesting that their transcription can be regulated with higher efficiency. This genomic feature may contribute to the high yield characteristics of *O. polymorpha*.

Besides the high similarity of genomic organization, the rDNAs of *O. polymorpha* HU-11/CBS4732 and *S. cerevisiae* share high nt sequence identities of 95.3% (1720/1805), 96.2% (152/158), 92% (3,111/3,381), and 96.7% (117/121) for 18S, 5.8S, 25S, and 5S rDNAs, respectively. These identities are little lower than those of *O. polymorpha* NCYC495 and DL-1, indicated that the rDNA genes are more conservative than the protein-coding genes. Therefore, rDNA is an important genomic feature for the detection, identification, classification and phylogenetic analysis of *Ogataea* spp. Unexpectedly, we found that the rDNAs (GenBank: FN392325) of *O. polymorpha* HU-11/CBS4732 and *P. pastoris* GS115 have nt sequence identities of 87.3% (1477/1691), 80% (84/105), and 80.5% (2,073/2,576) for 18S, 5.8S, and 25S rDNAs, respectively, which are much lower than those of *S. cerevisiae*. These results are not consistent with those of a previous study (Ravin et al., 2013), in which phylogenetic analysis using 153 protein-coding genes showed that *O. polymorpha* and *Pichia pastoris* GS115 are members of a clade that is distinct from the one that *S. cerevisiae* belongs to. Based on the previous study, HU-11/CBS4732 is phylogenetically closer to *P. pastoris* GS115 than *S. cerevisiae*. The nt sequence identities of rDNAs between HU-11/CBS4732 and *P. pastoris* GS115 are supposed to be higher than those between HU-11/CBS4732 and *S. cerevisiae*.

### Long Terminal Repeat Retrotransposons

**FIGURE 4:**
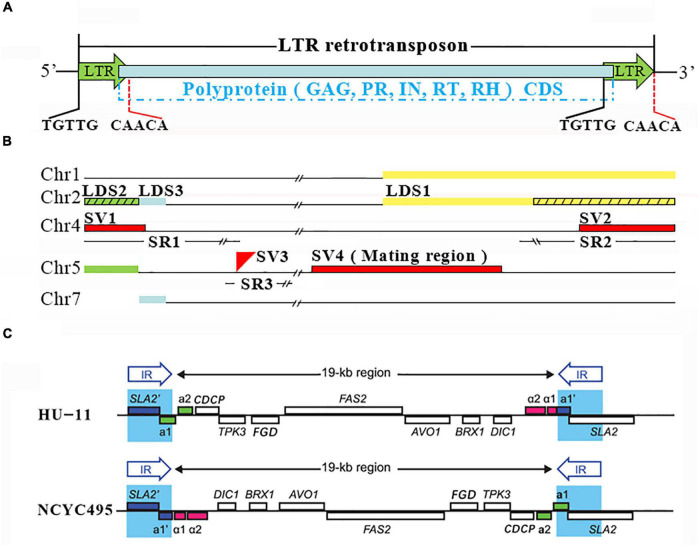
Long terminal repeat (LTR) retrotransposons, large duplicated segments and structural variations. **(A)** NCYC495 and HU-11/CBS4732 share identical 322-bp LTRs, which are flanked by TCTTG and CAACA at their 5′ and 3′ ends. Three of seven LTR-rts of HU-11/CBS4732 have no homologs in the NCYC495 genome due to misassembly. A LTR-rt consists of 5′ LTR, 3′ LTR and a single open reading frame (ORF) encoding a putative polyprotein. This polyprotein, if translated, can be processed into trunculated gag (GAG), protease (PR), integrase (IN), reverse transcriptase (RT) and RNase H (RH). **(B)** Chr1, 2, 4, 5, and 7 represent the chromosomes (GenBank: CP073033, 34, 36, 37, and 39) of the HU-11/CBS4732 genome. Three large duplicated segments (LDSs) named LDS1 (in yellow color), 2 (in green color) and 3 (in blue color) are supposed to be included in both NCYC495 and HU-11/CBS4732 genomes. However, LDS2 and a 14,090 bp part of LDS1 (indicated by black slashs) were not assembled into chromosome 2 of the NCYC495 genome. The genomic differences between the HU-11/CBS4732 and NCYC495 only include three structural variations (SVs), named SV1, 2 and 3 (in red color). The three SVs are located in three large syntenic regions (SRs) of HU-11/CBS4732, NCYC495, and DL-1 genomes with very high nt sequence identities, named SR1, 2 and 3. SV4 is a 22.6-Kb DNA region which functions in the determination of the yeast mating-type (MAT). **(C)** The graphic elements used to represent the genomes and genes were originally used in the previous study (Hanson et al., 2017). The HU-11 genome (GenBank: CP073033-40) contains a 22.6-Kb MAT region where MATα can be transcribed, while the NCYC495 genome (RefSeq: NW_017264698-704) contains an identical 22.6-Kb MAT region where MATa can be transcribed.

LTRs with lengths of 322 bp were discovered in all seven chromosomes of HU-11/CBS4732. These LTRs with the low GC content of 29% (94/322) are flanked by TCTTG and CAACA at their 5′ and 3′ ends (Figure 4A). In HU-11/CBS4732, a total of 14 LTRs are present in seven copies of intact LTR-rts (Supplementary File 1), which were identified as components of Tpa5 LTR-rts (GenBank: AJ439553) from *Pichia angusta* CBS4732 (a former name of *O. polymorpha* CBS4732) in a previous study (Neuveglise et al., 2002). A LTR-rt consists of 5′ LTR, 3′ LTR, and a single open reading frame (ORF) encoding a putative polyprotein (Figure 4A). This polyprotein, if translated, can be processed into truncated Gag (GAG), protease (PR), integrase (IN), reverse transcriptase (RT), and RNase H (RH). Based on the gene order (PR, IN, RT, and RH), the LTR-rts of HU-11/CBS4732 were classified into the Ty5 type of the Ty1/copia group (Ty1, 2, 4, and 5 types) (Agrawal and Ganley, 2018).

With the length corrected from 4,883 bp to 4,882 bp, a sequence (GenBank: AJ439558) was used as the reference of Tpa5 LTR-rts to search for homologs. The results confirmed that HU-11/CBS4732 is phylogenetically closest to NCYC495 and they share identical LTRs. However, the 322-bp LTRs of HU-11/CBS4732 and NCYC495 are quite distinct from the 282-bp LTRs of DL-1, which were reported as 290-bp solo LTRs in the previous study (Ravin et al., 2013). In addition, the amino acid (aa) sequences of the polyprotein with the length of 1417 aa in HU-11/CBS4732 and NCYC495 LTR-rts are distinct from those in DL-1. Based on the records in the UniProt Knowledgebase (UniProtKB), *O. polymorpha* strains DL-1, ATCC26012, BCRC20466, JCM22074, and NRRL Y-7560 have nearly the same aa sequences (UniProt: W1QI12) of the polyprotein. The above results suggest that the LTR-rt is another important genomic feature for the detection, identification, classification and phylogenetic analysis of *Ogataea* spp. Using RNA-seq data of NCYC495 (SRA: SRP124832), we discovered that the genes encoding the polyproteins in the LTR-rts of *O. polymorpha* are transcribed. If these putative polyproteins can be translated merits further studies.

In the previous study, 50,000 fragments of 13 *Hemiascomycetes* species were used to identify LTR-rts. However, the analysis was probably biased as it was based on only random sequences (approximately 1 kb on average) without the related genome information (Neuveglise et al., 2002). In the present study, seven copies of intact LTR-rts (As described above) were precisely located in the HU-11/CBS4732 genome (Figure 2A), with five in the sense strands of chromosome 1, 2, 3, 6, and 7 (named LTR-rt1, 2, 3, 6, and 7) and two in the antisense strands of chromosome 5 and 7 (named LTR-rt5R and 7R). LTR-rt1, 3, and 6 share very high nt identities of 99.9% with each other. LTR-rt1 or 3 contains a single ORF encoding a polyprotein with the same aa sequence, while LTR-rt6 contains a single ORF with a 42-bp insertion (encoding RSSLFDVPCSPTVD), compared to LTR-rt1 and 3. LTR-rt2, 5R, 7, and 7R contain several single nucleotide polymorphisms (SNPs), small insertions and deletions (InDels), which break the single ORFs into several ORFs. Genome comparison revealed that the homologs of LTR-rt2, 3, and 5R in HU-11/CBS4732 are present in the NCYC495 genome with very high nt identities of 99.9%, while the homologs of LTR-rt1, 7, and 7R, however, are absent in the NCYC495 genome. Further analysis determined that their absence in the NCYC495 genome resulted from misassembly.

### Large Duplicated Segments in Subtelomeric Regions

Syntenic comparison revealed that *O. polymorpha* HU-11/CBS4732 is so phylogenetically close to NCYC495 that the syntenic regions cover nearly 100% of their genomes, however, HU-11/CBS4732 and NCYC495 are significantly distinct from DL-1. HU-11/CBS4732 and NCYC495 share a nt identity of 99.5% through their whole genomes, including the rDNA regions and LTR-rts. In contrast, HU-11/CBS4732 and DL-1 share a comparatively low nt identity (<95%). Subsequently, the detection of structural variations (SVs) was performed between the HU-11/CBS4732 and NCYC495 genomes. Further analysis revealed that most of detected SVs are errors in the assembly of NCYC495 genome (Figure 4B), particularly including: (1) LTR-rt1, 7, and 7R (absent in NCYC495) which need be included in the NCYC495 genome; (2) two large deletions (absent in NCYC495) which need be added at 5′ and 3′ ends of chromosome 2 of NCYC495; and (3) an over-assembled large segment (absent in HU-11/CBS4732) at 3′ end of chromosome 6 (NW_017264698:1509870-1541475), which need be removed from chromosome 6 of NCYC495. Before the correction of above errors, (1), (2), and (3) were confirmed by long PacBio subreads. Particularly, (3) was confirmed as the telomeric region at the 3′ end of chromosome 6, which was wrongly assembled as the junction region at 5′ end of the over-assembled segment in the previous study and the reason is that the copy number of TRs [GGCGGGGT]_*n*_ (NW_017264698:1509840-1509869) in this telomeric region was under-estimated using short sequencing data.

Two large deletions [one type of SV (Zhang et al., 2016)] in the NCYC495 genome (As described above) are “false-positive” SVs caused by the misassembly of LDSs (Supplementary File 1) in the subtelomeric regions. In contrast, all LDSs were correctly assembled in the HU-11/CBS4732 genome. Using long (>30 Kb) PacBio subreads, human curation (see section “Materials and Methods”) was performed to verify the locations of the LDSs, particularly three LDSs named LDS1, 2 and 3 with lengths of ∼27,850, ∼5,100, and ∼2,500 bp, respectively (Figure 4B): (1) LDS1 and its paralog are present at 3′ ends of chromosome 2 and 1 in the HU-11/CBS4732 genome, respectively, while the paralog of LDS1 was correctly assembled into 3′ end of chromosome 1 of NCYC495, but a 14,090 bp part of LDS1 was not assembled into 3′ end of chromosome 2, which corresponds to a large deletion; and (2) LDS2 and its paralog are present at 5′ ends of both chromosomes 2 and 5 in the HU-11/CBS4732 genome, while the paralog of LDS2 was correctly assembled into 5′ end of chromosome 5, but LDS2 was not assembled into 5′ end of chromosome 2 of NCYC495, which corresponds to the other large deletion. Different from LDS1 and LDS2, LDS3 and its paralog were correctly assembled in the NCYC495 genome. LDS3 is downstream of LDS2 in chromosome 2, and the paralog of LDS3 is present at 5′ end of chromosome 7 (Figure 4B). LDS1 and 2 had not been discovered before the present study, mainly because they are nearly identical to their paralogs. Particularly, there are only four mismatches and one 1-bp gap between LDS1 and its paralog. As an important finding, telomeric TR-like sequences [ACCCCGCC]_*n*_ or [ACCCGCC]_*n*_ (n > 2) were discovered at 3′ ends of LDS2 and its paralog (located on both chromosomes 2 and 5), and at 3′ end of LDS3′s paralog (located in chromosome 7). The discovery of these sequences (Supplementary File 1) indicated that these LDSs were integrated at 5′ ends of the old telomeric regions by their 3′ ends.

### Structural Variations Between HU-11/CBS4732 and NCYC495

The major errors in the assembly of NCYC495 genome (RefSeq: NW_017264698-704) include incomplete rDNAs, misassembly of LTR-rts, under-assembly of LDSs and over-assembly of a large segment. After we corrected these errors using the syntenic regions, only four SVs between HU-11/CBS4732 and NCYC495 remained and were named as SV1, SV2, SV3, and SV4 (Figure 4B). Further analysis revealed that SV4 is a very special “false-positive” SV. Actually, SV4 is a 22.6-Kb DNA region (Figure 4C) which functions in the determination of the yeast mating-type (MAT). According to previous studies (Hanson et al., 2017), yeast mating generally occurs between two haploid cells with opposite genotypes (MATa and MATα) at this locus, to form a diploid zygote (MATa/α). *Ogataea* spp. contain both a MATa locus and a MATα locus in chromosome 5, approximately 19 Kb apart (Figure 4C). The two MAT loci are beside two copies of an identical 2-Kb DNA sequence, which form two inverted repeats (IRs). During MAT switching, the two copies of the IR recombine, inverting the orientation of the 19-Kb region relative to the rest of the chromosome. The MAT locus proximal to the centromere is not transcribed, probably due to silencing by centromeric heterochromatin, whereas the distal MAT locus is transcribed. The HU-11 genome contains a 22.6-Kb MAT region (MAT-HU11) where MATα can be transcribed, while the NCYC495 genome contains a 22.6-Kb MAT region (MAT-NCYC495) where MATa can be transcribed. There is only one 1-bp gap between the large segments MAT-HU11 and the reverse-complimentary sequence of MAT-NCYC495 (Supplementary File 1). Therefore, the HU-11 (GenBank: CP073033-40) and NCYC495 (RefSeq: NW_017264698-704) genomes represent genomes of *O. polymorpha* MATα and MATa cells, respectively. MAT regions can’t be used as a genomic marker to characterize different *O. polymorpha* strains, as MAT switching can be induced without environmental signals. For example, we found that MAT switching of HU-11 even occur under optimal growth conditions (pH = 5.5, *T* = 32°C), although the frequency is extremely low (1/264).

Only three SVs (SV1, SV2, and SV3) are true-positive. SV1 and SV2 are present at 5′ and 3′ ends of chromosome 4, respectively, while the location of SV3 is close to 5′ ends of chromosome 5 (Figure 4C). Five sequences involved in these three SVs are SV1-CBS4732 and SV2-CBS4732 in the HU-11/CBS4732 genome and SV1-NCYC495, SV2-NCYC495, and SV3-NCYC495 in the NCYC495 genome (Supplementary File 1). These five sequences can be used to identify *O. polymorpha* strains, particularly CBS4732, NCYC495, and their derivative strains. Blasting the five sequences to the NCBI NT database, we found that SV1-CBS4732 and SV2-NCYC495 are nearly identical (>98%) to their orthologs at 5′ and 3′ ends of chromosome 4 in the DL-1 genome (GenBank: CP080319), respectively, while SV1-NCYC495 and SV2-CBS4732 have no homologs in chromosome 4 of DL-1. As an insertion into the NCYC495 genome, SV3-NCYC495 has a very high nt sequence identity (>91%) to its homolog in the DL-1 genome. Further analysis showed that the three SVs are located in three large syntenic regions (SRs) of HU-11/CBS4732, NCYC495, and DL-1 genomes with very high nt sequence identities (>95%). Three SRs are: (1) SR1 with a length of 161,844 bp at 5′ ends of chromosome 4; (2) SR2 with a length of 81,748 bp at 3′ ends of chromosome 4; and (3) SR3 with a length of 11,087 bp close to 5′ ends of chromosome 5. The above results revealed that many recombination events occurred in chromosome 4 and 5 of CBS4732 and NCYC495′ ancestors after their divergence, particularly: (1) recombination events occurred at 5′ end of chromosome 4 of the NCYC495′ ancestor, resulting in the acquisition of SV1-NCYC495; (2) recombination events occurred at 3′ end of chromosome 4 of the CBS4732′ ancestor, resulting in the acquisition of SV2-CBS4732; (3) recombination events occurred close to 5′ end of chromosome 5 of the CBS4732′ ancestor, resulting in the loss of SV3-CBS4732 (the hypothetical homolog of SV3-NCYC495).

Only a few genes (predicted as 25) were involved in the three SVs between HU-11/CBS4732 and NCYC495 (Table 2). Among the 25 genes (Table 2), 10 genes (OGAPO_03766-67 and OGAPO_00003-08) of HU-11/CBS4732 and 11 genes (OGAPODRAFT_24127, 16381, 24129, 12876, 16382, 76936, 16706, 37951, 93168, 75778, and 75779) of NCYC495 have no orthologs in NCYC495 and HU-11/CBS4732, respectively and two genes (OGAPODRAFT_13497 and 15973) in NCYC495 were significantly changed into two other ones (OGAPO_13497 and 15973) in HU-11/CBS4732, resulting in different aa sequences. Blasting the proteins encoded by these 25 genes (Supplementary File 1) to the UniProt database, we found that the proteins encoded by five genes (OGAPODRAFT_24127, 16381, 24129, 12876, and 16382) in SV1-NCYC495 have the highest sequence similarities to their homologs encoded by six genes (HPODL_02401, 02402, 02403, 02404, 02405, and 02398) at 3′ end of chromosome 6 (RefSeq: NC_027860) in the DL-1 genome. The above results suggest that SV1-NCYC495 from chromosome 6 of NCYC495′ ancestor was acquired by chromosome 4 of NCYC495 *via* translocation. The proteins encoded by six genes (OGAPO_00003-08) in a major part (more than 80%) of SV2-CBS4732 have no homologs in *Ogataea polymorpha* NCYC495 or DL-1, but have the highest sequence similarities to their homologs in *O. thermophila*, followed by *O. philodendri* and *O. haglerorum*. These six proteins also have homologs in the *Cyberlindnera jadinii* strain NRRL Y-1542. Furthermore, we found that the proteins encoded by two genes (OGAPO_00001-02) in the minor part of SV2-CBS4732 (from chromosome 4) have the highest sequence similarities to their homologs encoded by genes in other chromosomes. These findings revealed more combination events occurred between chromosome 4 and other chromosomes within the genome of NCYC495′ ancestor or CBS4732′ ancestor.

**TABLE 2:** Twenty five different genes between HU-11/CBS4732 and NCYC495.

Gene	Locus	Orthologs (CBS4732/NCYC495/DL-1)	Function
OGAPO_03767	SV1- CBS4732	OGAPO_03767/-/HPODL_03767	12-oxophytodienoate reductase 3 (OPR3)
OGAPO_03766	SV1- CBS4732	OGAPO_03766/-/HPODL_03766	Aminotriazole resistance protein
OGAPODRAFT_24127	SV1-NCYC495	-/OGAPODRAFT_24127/-	Myo-inositol transporter 1
OGAPODRAFT_16381	SV1-NCYC495	-/OGAPODRAFT_16381/-	Aldo keto reductase (ARK)
OGAPODRAFT_24129	SV1-NCYC495	-/OGAPODRAFT_24129/-	Amidase
OGAPODRAFT_12876	SV1-NCYC495	-/OGAPODRAFT_12876/-	MFS transporter
OGAPODRAFT_16382	SV1-NCYC495	-/OGAPODRAFT_16382/-	NADP-dependent alcohol dehydrogenase 6
OGAPO_00001	SV2- CBS4732	OGAPO_00001/-/-	Aminotriazole resistance protein
OGAPO_00002	SV2- CBS4732	OGAPO_00002/-/-	Aryl-alcohol dehydrogenase
OGAPO_00003	SV2- CBS4732	OGAPO_00005/-/-	Sterol regulatory element-binding protein ECM22
OGAPO_00004	SV2- CBS4732	OGAPO_00007/-/-	Agmatine ureohydrolase
OGAPO_00005	SV2- CBS4732	OGAPO_00008/-/-	P-loop containing nucleoside triphosphate hydrolase protein
OGAPO_00006	SV2- CBS4732	OGAPO_00009/-/-	MFS general substrate transporter
OGAPO_00007	SV2- CBS4732	OGAPO_00010/-/-	Acetylornithine aminotransferase, mitochondrial (ARG8)
OGAPO_00008	SV2- CBS4732	OGAPO_00012/-/-	Aldo keto reductase (ARK)
OGAPODRAFT_13497*	SV2-NCYC495	OGAPO_13497/*/HPODL_00892	Basic amino-acid permease
OGAPODRAFT_76936	SV2-NCYC495	-/OGAPODRAFT_76936/HPODL_00891	Transcriptional activator protein DAL81
OGAPODRAFT_16706	SV2-NCYC495	-/OGAPODRAFT_16706/HPODL_00890	DUF1479-domain-containing protein
OGAPODRAFT_37951	SV2-NCYC495	-/OGAPODRAFT_37951/HPODL_02394	MFS domain-containing protein
OGAPODRAFT_93168	SV3-NCYC495	-/OGAPODRAFT_93168/HPODL_04518	MFS domain-containing protein
OGAPODRAFT_15973*	SV3-NCYC495	OGAPO_15973/*/HPODL_04520	MFS sugar transporter
OGAPODRAFT_75778	SV3-NCYC495	-/OGAPODRAFT_75778/HPODL_04517	Adenosine deaminase
OGAPODRAFT_75779	SV3-NCYC495	-/OGAPODRAFT_75779/HPODL_04516	Zn(2)-C6 fungal-type domain-containing protein

The genomic differences between Ogataea polymorpha HU-11/CBS4732 and NCYC495 include SNPs, small InDels, and only three SVs (Figure 4B). Five sequences (SV1-CBS4732, SV2-CBS4732, SV1-NCYC495, SV2-NCYC495, and SV3-NCYC495) were involved in these three SVs. Only 25 genes (Supplementary File 1) were involved in the three SVs between HU-11/CBS4732 and NCYC495. 10 genes (OGAPO_03766-67 and OGAPO_00003-08) of HU-11/CBS4732 and 11 genes (OGAPODRAFT_24127, 16381, 24129, 12876, 16382, 76936, 16706, 37951, 93168, 75778, and 75779) of NCYC495 have no orthologs in NCYC495 and HU-11/CBS4732, respectively. *Two genes (OGAPODRAFT_13497 and 15973) in NCYC495 were significantly changed into two other ones (OGAPO_13497 and 15973) in HU-11/CBS4732. Six genes (OGAPO_00003-08) in a major part of SV2-CBS4732 have no homologs in NCYC495 or DL-1. OGAPO_, OGAPODRAFT_, and HPODL_ are prefix of gene IDs of HU-11/CBS4732, NCYC495, and DL-1, respectively.

## Conclusion

The *O. polymorpha* strain CBS4732 *ura3*Δ (named HU-11) is a nutritionally deficient mutant derived from CBS4732 by a 5-bp insertion of “GAAGT” into the 32nd position of the *URA3* CDS; this insertion causes a frame-shift mutation of the *URA3* CDS, resulting in the loss of the *URA3* functions. Since the difference between the genomes of CBS4732 and HU-11 is only five nts, HU-11 has the same reference genome as CBS4732 (noted as HU-11/CBS4732). In the present study, we have assembled the full-length genome of *O. polymorpha* HU-11/CBS4732 using high-depth PacBio and Illumina data. 5′ and 3′ telomeric, subtelomeric, rDNA, LTR-rts, low complexity, and other repeat regions were curated to improve the genome quality. In brief, the main findings include complete rDNAs, complete LTR-rts, three LDSs in subtelomeric regions and three SVs between the HU-11/CBS4732 and NCYC495 genomes. SV1, LDS1, LDS2, and LDS3 were validated using the strand-specific RNA-seq data of NCYC495 (SRA: SRP124832). These findings are very important for the assembly of full-length genomes of yeast and the correction of assembly errors in the published genomes of *Ogataea* spp.

The present study preliminarily revealed the relationship between *O. polymorpha* CBS4732, NCYC495, and DL-1. HU-11/CBS4732 is so phylogenetically close to NCYC495 that the syntenic regions cover nearly 100% of their genomes. Moreover, HU-11/CBS4732 and NCYC495 share a nucleotide identity of 99.5% through their whole genomes, including the rDNA regions and LTR-rts. The genomic differences between HU-11/CBS4732 and NCYC495 include SNPs, small InDels, and only three SVs. CBS4732 and NCYC495 can be regarded as the same strain in basic research and industrial applications. The HU-11 (GenBank: CP073033-40) and NCYC495 (RefSeq: NW_017264698-704) genomes represent genomes of *O. polymorpha* MATα and MATa cells, respectively. Large segments SV1-CBS4732, SV2-CBS4732, SV1-NCYC495, SV2-NCYC495, and SV3-NCYC495 involved in the three SVs can be used to identify *O. polymorpha* strains, particularly CBS4732, NCYC495, and their derivative strains. Among these five large segments, SV2-CBS4732 merits further investigation. The proteins encoded by six genes in a major part of SV2-CBS4732 have no homologs in *Ogataea polymorpha* NCYC495 or DL-1, but have the highest sequence similarities to their homologs in *O. thermophila*, followed by *O. philodendri* and *O. haglerorum*. These six proteins also have homologs in the *Cyberlindnera jadinii* strain NRRL Y-1542. As most genome sequences do not contain complete subtelomeric regions where the six genes locate, the origin of these genes are still not determined.

Only with the exact sequences of subtelomeric regions, can we discover the SV1 and SV2. Furthermore, we reported for the first time LDSs in the subtelomeric regions of *Ogataea* genomes. LDS1 and LDS2 had not been discovered before the present study, mainly because they are nearly identical to their paralogs. A computational study (Brown, 2010) showed that subtelomeric gene families are evolving and expanding much faster than gene families which do not contain subtelomeric genes in yeasts. This previous study also concluded that the extraordinary instability of eukaryotic subtelomeres supports rapid adaptation to novel niches by promoting gene recombination and duplication followed by the functional divergence of the alleles. Our results indicated that large segment duplication in subtelomeric regions occurs in a size to the extent of ∼27,850 bp and suggests that the genome expansion in methylotrophic yeasts is mainly driven by large segment duplication in subtelomeric regions. The discovery of telomeric TR-like sequences at 3′ ends of the LDSs indicated that they were integrated at 5′′ ends of the old telomeric regions by their 3′ ends. The exact LDS and telomeric TR-like sequences are very important for the investigation of the molecular mechanism (if *via* recombination or not) that underlies large segment duplication in subtelomeric regions.

## Materials and Methods

The *Ogataea polymorpha* strain HU-11 (CGMCC No. 1218) was preserved in the China General Microbiological Culture Collection Center (CGMCC). DNA extraction and quality control were performed as described in our previous study (Wang Y. et al., 2016). A 500 bp DNA library was constructed as described in our previous study (Wang Y. et al., 2016) and sequenced on the Illumina HiSeq X Ten platform. A 10 Kb DNA library was constructed and sequenced on the PacBio Sequel platforms, according to the manufacturer′s instruction. The cleaning and quality control of PacBio data was performed with the software SMRTlink v5.0 (–minLength = 50, –minReadScore = 0.8), while the cleaning and quality control of Illumina data was performed with the software Fastq_clean (Zhang et al., 2014) v2.0. PacBio data was used to assemble the HU-11/CBS4732 draft genome with the software MECAT (Xiao et al., 2016) v1.2. To polish the draft genome, the software BWA was used to align Illumina data to the HU-11/CBS4732 draft genome. Then, the software samtools was used to obtain the BAM and pileup files from the alignment results. Perl scripts were used to extract the consensus sequence from the pileup file. This polishing procedure was repeatedly performed until human curation started. The reference genomes of *O. polymorpha* HU-11/CBS4732, NCYC495 and DL-1 are available at the NCBI GenBank or RefSeq database under the accession numbers CP073033-40, NW_017264698-704 and NC_027860-66. Another genome of *O. polymorpha* DL-1 (GenBank: CP080316-22) was also used for syntenic comparison and SV detection, as this genome is more complete than the genome (RefSeq: NC_027860-66).

The genome sequences of 34 species in the *Ogataea* or *Candida* genus were downloaded from the Genome-NCBI datasets and their accession numbers were included in Supplementary File 1. Syntenic comparison of genomes was performed using the CoGe website,^1^ only for visualization. The detailed syntenic comparison and SV detection were performed locally using the software blast v2.9.0 and Perl scripts. Using the software IGV (Thorvaldsdóttir et al., 2013) v2.0.34, human curation of the poly(GC) regions, 5′ and 3′ telomeric, subtelomeric, rDNA, LTR-rts, low complexity, and other repeat regions was performed with 103,345 long (>20 Kbp) PacBio subreads. The curation criteria is: (1) the junctions between large segments (e.g., LTR-rts, LDSs, or SVs) must be spanned by a long PacBio subread; and (2) the corrected nucleotides must be confirmed by more than 5 long PacBio subreads. To estimate the frequency of MAT switching, each long PacBio subread was counted with human curation. Statistical computation and plotting were performed using the software R v2.15.3 with the Bioconductor packages (Gao et al., 2014). Prediction of protein-coding genes (>150 bp) was performed using the software AUGUSTUS (Hoff and Stanke, 2013) v2.7.0. Strand-specific RNA-seq data (SRA: SRP124832) was used to curate gene annotations of HU-11/CBS4732, NCYC495 and DL-1. As the reads in the data SRP124832 correspond to the reverse-complementary counterpart of transcripts, they were transformed into their reverse-complementary sequences for all the analyses in the present study.

## Data Availability Statement

The complete genome sequence of the *O. polymorpha* HU-11/CBS4732 is available at the NCBI GenBank database under the accession number CP073033-40, in the project PRJNA687834.

## Author Contributions

SG conceived the project and drafted the manuscript. SG and DW supervised the present study. JC assembled the HU-11/CBS4732 genome, prepared the figures, tables, and Supplementary Material. JB and HF executed the experiments. SG, QS, and TY analyzed the data. SG, HW, WB, and JR revised the manuscript. All authors have read and approved the manuscript.

## Conflict of Interest

HW was employees by Tianjin Hemu Health Biotechnological Co., Ltd. The remaining authors declare that the research was conducted in the absence of any commercial or financial relationships that could be construed as a potential conflict of interest.

## Publisher’s Note

All claims expressed in this article are solely those of the authors and do not necessarily represent those of their affiliated organizations, or those of the publisher, the editors and the reviewers. Any product that may be evaluated in this article, or claim that may be made by its manufacturer, is not guaranteed or endorsed by the publisher.

## Footnotes

1
https://genomevolution.org/CoGe

## Abbreviations

TR: tandem repeat
STR: short tandem repeat
LTR: long terminal repeat
ORF: open reading frame
CDS: coding sequence
SV: structural variation
SNP: single nucleotide polymorphism
InDel: insertion and deletion
mt: mitochondrial
nt: nucleotide
aa: amino acid.
